# Management adaptation of invertebrate fisheries to an extreme marine heat wave event at a global warming hot spot

**DOI:** 10.1002/ece3.2137

**Published:** 2016-04-24

**Authors:** Nick Caputi, Mervi Kangas, Ainslie Denham, Ming Feng, Alan Pearce, Yasha Hetzel, Arani Chandrapavan

**Affiliations:** ^1^ Western Australian Fisheries and Marine Research Laboratories PO Box 20 North Beach Western Australia 6920 Australia; ^2^ CSIRO Oceans and Atmosphere Private Bag No. 5 Wembley Western Australia 6913 Australia; ^3^ Curtin University GPO Box U1987 Perth Western Australia 6845 Australia; ^4^ School of Civil, Environmental, and Mining Engineering and The UWA Oceans Institute (M470) University of Western Australia 35 Stirling Highway Crawley Western Australia 6009 Australia

**Keywords:** Climate change, crabs, environmental effects, prawns, pre‐recruit, scallops, stock‐recruitment, water temperature

## Abstract

An extreme marine heat wave which affected 2000 km of the midwest coast of Australia occurred in the 2010/11 austral summer, with sea‐surface temperature (SST) anomalies of 2–5°C above normal climatology. The heat wave was influenced by a strong Leeuwin Current during an extreme *La Niña* event at a global warming hot spot in the Indian Ocean. This event had a significant effect on the marine ecosystem with changes to seagrass/algae and coral habitats, as well as fish kills and southern extension of the range of some tropical species. The effect has been exacerbated by above‐average SST in the following two summers, 2011/12 and 2012/13. This study examined the major impact the event had on invertebrate fisheries and the management adaption applied. A 99% mortality of Roei abalone (*Haliotis roei*) and major reductions in recruitment of scallops (*Amusium balloti*), king (*Penaeus latisulcatus*) and tiger (*P. esculentus*) prawns, and blue swimmer crabs were detected with management adapting with effort reductions or spatial/temporal closures to protect the spawning stock and restocking being evaluated. This study illustrates that fisheries management under extreme temperature events requires an early identification of temperature hot spots, early detection of abundance changes (preferably using pre‐recruit surveys), and flexible harvest strategies which allow a quick response to minimize the effect of heavy fishing on poor recruitment to enable protection of the spawning stock. This has required researchers, managers, and industry to adapt to fish stocks affected by an extreme environmental event that may become more frequent due to climate change.

## Introduction

A marine heat wave event is defined as a prolonged discrete anomalously warm water event that can be described by its duration, intensity, rate of evolution, and spatial extent (Pearce and Feng 2013; Hobday et al. 2016). Extreme water temperatures have significant impacts on marine biology and fisheries around the globe, and there have been increasing frequency of marine heat waves at various regions; however, the implications of the marine heat waves on fisheries management have not been fully assessed.

**Figure 1:**
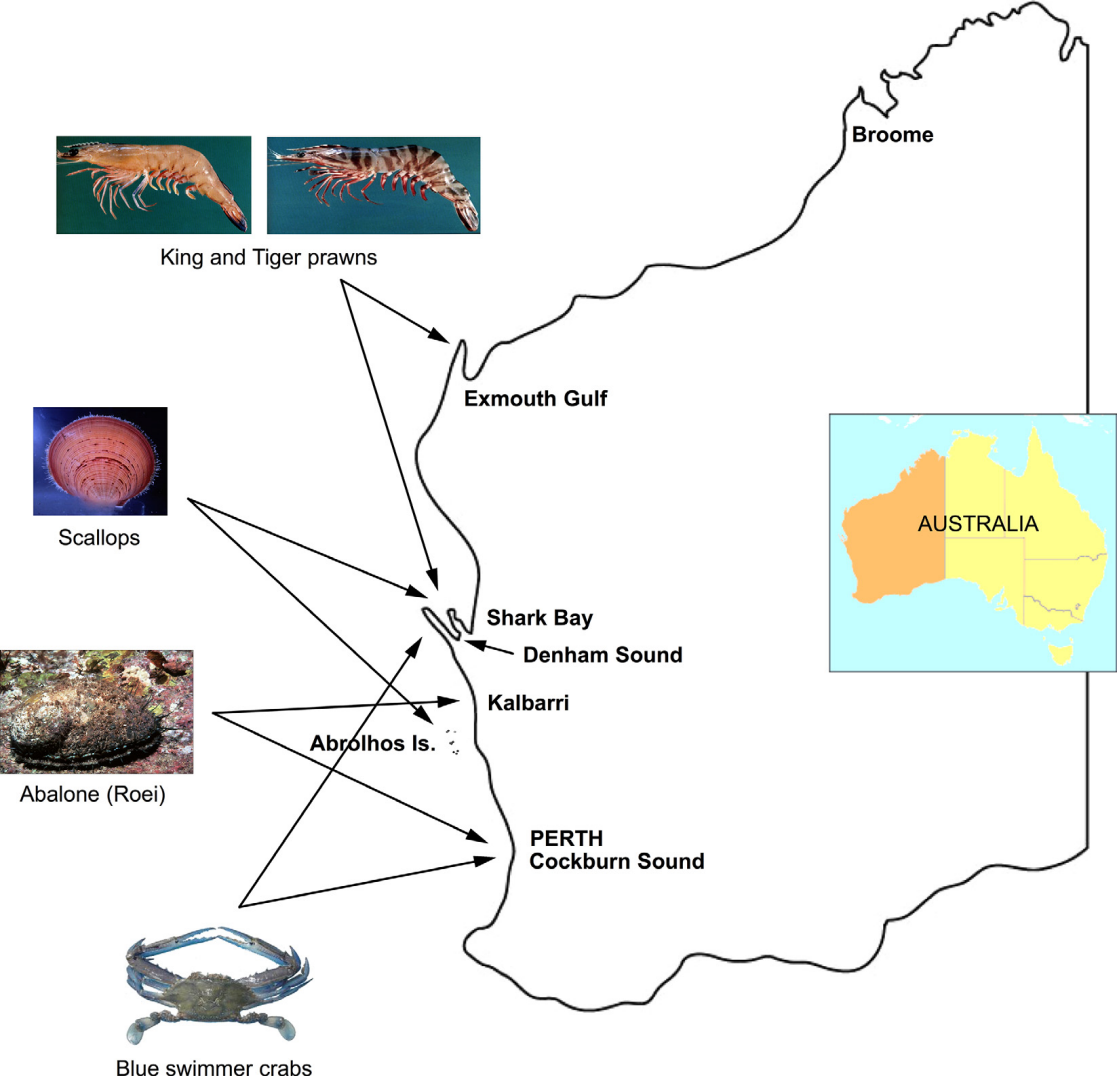
Map of Western Australia showing key locations and location of fisheries.

A marine heat wave affected 2000 km of the Western Australian coast, centered at the midwest coast during the 2010/11 austral summer (Fig. 1) (Pearce and Feng 2013; Wernberg et al. 2013; Caputi et al. 2014b). Water temperatures exceeded 5°C above the average with warming anomalies of 2–4°C persisting for more than 10 weeks along the coast (Wernberg et al. 2013). The heat wave was influenced by a strong Leeuwin Current during an extreme *La Niña* event and an anomalously high heat flux (Feng et al. 2013; Pearce and Feng 2013). Feng et al. (2013) described the heat wave as a unique Ningaloo *Niño* event as a result of an alignment of intraseasonal to interdecadal processes, which resulted in an earlier surge of the Leeuwin Current during austral summer which was associated with high water temperatures.

Pearce and Feng (2007) identified the lower west coast of Australia as a hot spot of water temperature increases in the Indian Ocean with a 1°C increase over the past 40 years, particularly during the austral autumn/winter period (Caputi et al. 2009). It has also been classified as one of 24 global warming hot spots (Hobday and Pecl 2013). The summers of 2011/12 and 2012/13 also experienced above‐average water temperatures after the 2010/11 summer heat wave event (Caputi et al. 2014b), which have exacerbated the effect of the heat wave on the marine ecosystem. Wernberg et al. (2013) showed that biodiversity patterns of temperate seaweeds, sessile invertebrates, and demersal fish were significantly different after the 2010/11 warming event which led to a reduction in abundance of habitat‐forming seaweeds, a subsequent shift in community structure, and a southward distribution shift in tropical finfish communities. Seagrasses in Shark Bay (Fraser et al. 2014) and Exmouth Gulf were also significantly affected by the heat wave. Coral bleaching followed by high mortality was recorded in areas of Ningaloo and Abrolhos Is. (Depczynski et al. 2012).

This Western Australian heat wave event is one of several regions where large‐scale marine heat waves have occurred in recent years (Hobday et al. 2016). These heat waves can have a major effect on the marine ecosystem including shifts in species distribution, local extinctions, and significant effects on fisheries and aquaculture. An event in the northwestern Mediterranean rock benthic communities caused large‐scale mass mortality of benthic species over hundreds of kilometers in the summer of 1999 (Cerrano et al. 2000) and 2003 when macroinvertebrate species over several thousand kilometers of coastline were affected (Garrabou et al. 2009). The latter Mediterranean event appears to have been initiated by air temperatures 3–6°C above average. The Northwest Atlantic was affected by an ocean heat wave event in 2012 which was the largest, most intense event in this region for over 30 years. This resulted in a major impact on coastal ecosystems and economies (Mills et al. 2013). The northeast Pacific also experienced an unprecedented warming event in 2014 (Bond et al. 2015), and the ecological implications of the event are still being assessed. Under the present warming trend influenced by anthropogenic forcing, these extreme events are expected to become more common; therefore, it is important to assess the management adaptation response.

Under the influence of the West Australian marine heat wave, short‐term (<2 months) and longer‐term (>1 year) effects on invertebrate fisheries were observed (Caputi et al. 2014b). The short‐term effects were fish kills with the most significant event with nearly all of the Roei abalone (*Haliotis roei*) dying in the midwest of Western Australia. The main longer‐term impacts have been on the recruitment and survival of invertebrate species such as blue swimmer crabs (*Portunus armatus*) and scallops (*Amusium balloti*). The heat wave also had some positive and negative effects on the king (*Penaeus latisulcatus*) and tiger (*P. esculentus*) prawn stocks in different regions. An important question is whether the high water temperatures have had a direct impact during the spawning and larval phase or whether they have had an indirect effect on the habitat such as seagrass. The management focus on the fisheries affected by the heat wave has been to protect the spawning stock to try and avoid recruitment overfishing. Fisheries can typically collapse when heavy fishing pressure continues during years of poor recruitment.

This study examined the effect of the extreme heat wave on invertebrate fisheries in Western Australia and is one of the first to assess the management response to these events. The specific purpose was to examine the relationship between water temperature and recruitment of coastal invertebrate stocks as background to assessing the effect of the extreme environmental event. In particular, the water temperature increases during the spawning and larval and juvenile phase of a number of invertebrate fisheries such as blue swimmer crabs, scallops, king and tiger prawns, and abalone were examined as well as their management implications. The specific effect of the heat wave on the spawning stock of three scallop stocks and its effect on the potential recovery of the stocks were also examined.

## Materials and Methods

### Marine environment

The NOAA OIv2 sea‐surface temperature (SST) data from 1982 to June 2013 at ¼ degree (~28 km) resolution (Reynolds et al. 2007) were used to calculate monthly anomalies for each grid point relative to 1982–2012. The monthly mean values for February and June are used to represent summer and winter conditions. Temperature loggers were used to assess nearshore water temperatures (site details in Caputi et al. (2015)). Comparisons with temperature logger data showed that satellite SSTs were in general agreement within 1–2°C, thus indicating that satellite SST data can be reliably used to enable monitoring of water temperatures as also found by Smale and Wernberg (2009).

### Heat wave effect on fisheries

The effect of the warm temperatures during the summers of 2010/11 to 2012/13 on a number of invertebrate fisheries was undertaken using the correlation between the historical abundance indices for the fisheries and the monthly SST and at appropriate lags taking into account the time of spawning. The effect of spawning stock was also examined.

The abundance indices examined in the correlation assessment were as follows: (1) Shark Bay crabs: standardized fishers' catch rates; (2) Shark Bay scallops: recruit (0+) catch rates from the annual November survey; (3) Abrolhos Is. scallops: recruit catch rates (1 year old) in annual October survey; and (4) Exmouth Gulf and Shark Bay prawn and tiger prawn: recruit catch rates in annual March–April survey. The recruitment measures for prawn and scallops have generally been found to be reliable indicators as they have been shown to be good predictors of catch (Caputi et al. 2014a).

## Results

### Marine environment

**Figure 2:**
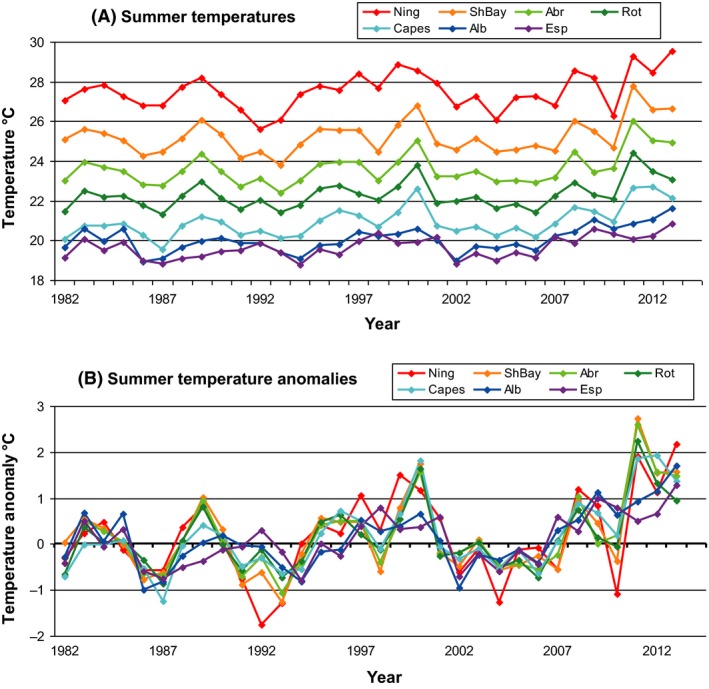
(A) Summer (December–February) sea‐surface temperature (SST) for 1^o^ blocks off Western Australia on the west coast near Ningaloo, Shark Bay, Abrolhos Is., Rottnest Is., and the Capes region, and the south coast near Albany and Esperance. (B) Summer SST anomalies for the above locations.

The summer SSTs off the west coast of Australia since 1982 highlighted the strength of the marine heat wave in 2011 along the west coast of Australia (Fig. 2). The water temperature was 2–3°C above the long‐term monthly average for all the west coast locations in early 2011 which represent the highest recorded (Hobday et al. 2016). The elevated temperatures persisted into the 2011/12 and 2012/13 summers. The 2010/11 heat wave developed off northwest Australia in November and moved toward the coast and extended south, reaching peak intensity along the midwest coast in February 2011 (Pearce and Feng 2013). Hourly temperature logger measurements from five coastal locations and the Abrolhos Is. over the past decade showed that peak daily temperature anomalies peaked at ~5°C above the long‐term average for short periods in late February and early March 2011 (Pearce and Feng 2013).

**Figure 3:**
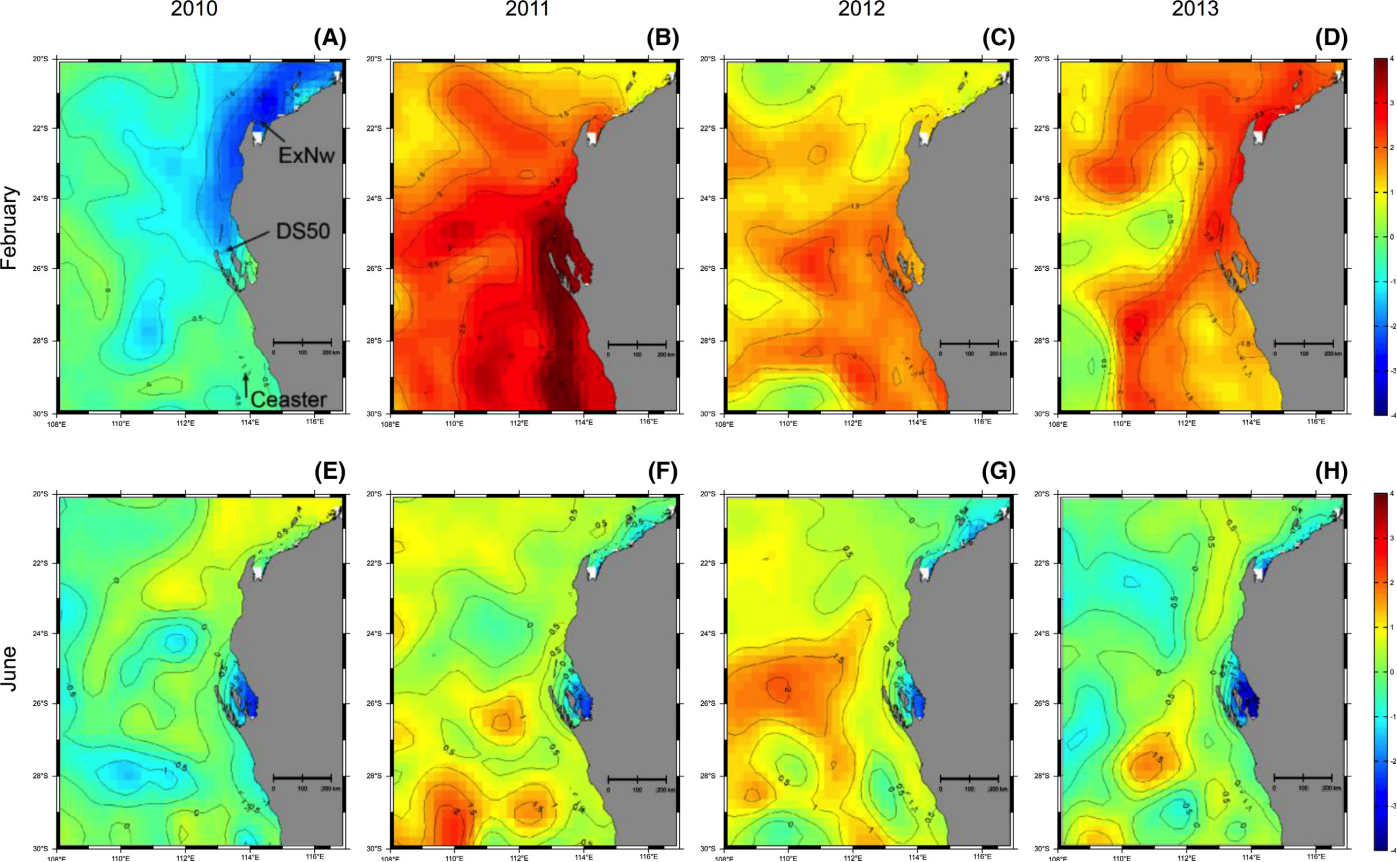
Monthly sea‐surface temperature (SST) anomalies relative to 1982–2012 mean temperatures based on the 28 km resolution OIv2 dataset for February (A–D) and June (E–H) and temperature contours are shown at 0.5°C intervals. The names in (A) represent SST locations in Exmouth Gulf (ExNw), Shark Bay (DS50), and Abrolhos Is. (Ceaster).

Ocean temperatures in 2010 were cooler from Shark Bay northward as influenced by the 2009/10 *El Niño*. SSTs were above average during the summers of 2010/11 to 2012/13 with below‐average winter water temperatures in the two major embayments of Exmouth Gulf and Shark Bay (Fig. 3). Shark Bay experienced the largest deviation from mean temperatures due to its enclosed geography and shallow depths which makes it more vulnerable to anomalous air–ocean heat fluxes. Thus, this region is not only a hot spot of global warming influence, but also a sensitive spot of interannual climate variability. Shark Bay had its highest SST during February 2011 but then experienced SST at 3°C *below* average during June 2013 (Fig. 3). At the peak of the heat wave in February 2011, the entire region from the Abrolhos to Exmouth experienced extremely high temperatures; however, the February SST anomalies showed the summer of 2012/13 to be warmer than 2010/11 in Exmouth Gulf (Fig. 3).

### Abalone fishery

An immediate impact of the heat wave on invertebrate fisheries was the catastrophic mortality of the abalone, *Haliotis roei,* in the midwest region, resulting in this fishery being closed and monitoring and restocking programs being implemented (Hart 2014). Mortality rates were location‐specific, with survival at one location estimated to be 0.01% with higher survival of 5–10% and 80–90% at other locations. These fish kills probably occurred due to a combination of record‐high water temperatures and very calm conditions (Pearce et al. 2011) causing deoxygenation of the water. The high mortality has made the natural recovery of this stock problematic, and therefore, restocking is being evaluated based on translocation and the release of hatchery‐grown abalone.

In the Perth fishery, which is south of the central area where the heat wave peaked, major mortalities were not observed; however, a stunting of the growth in mature age classes has resulted in a 30% drop in numbers of the sublegal cohort growing into the legal size‐class (A. Hart, pers. comm.).

### Shark Bay crab fishery

The blue swimmer crab (*Portunus armatus*) fishery in Shark Bay has become Australia's largest crab fishery, with peak landings of 828 *t* with two sectors fishing the crabs, trap fishers, and prawn trawlers who retain crabs as by‐product. However by late 2011, crab stocks were at record‐low levels with poor commercial catch rates and low survey catch rates in November 2011. Industry initiated the closure of the trap fishery and the trawl sector imposed voluntary nonretention of crabs. Trawl and trap surveys were undertaken to monitor the stock.

**Figure 4:**
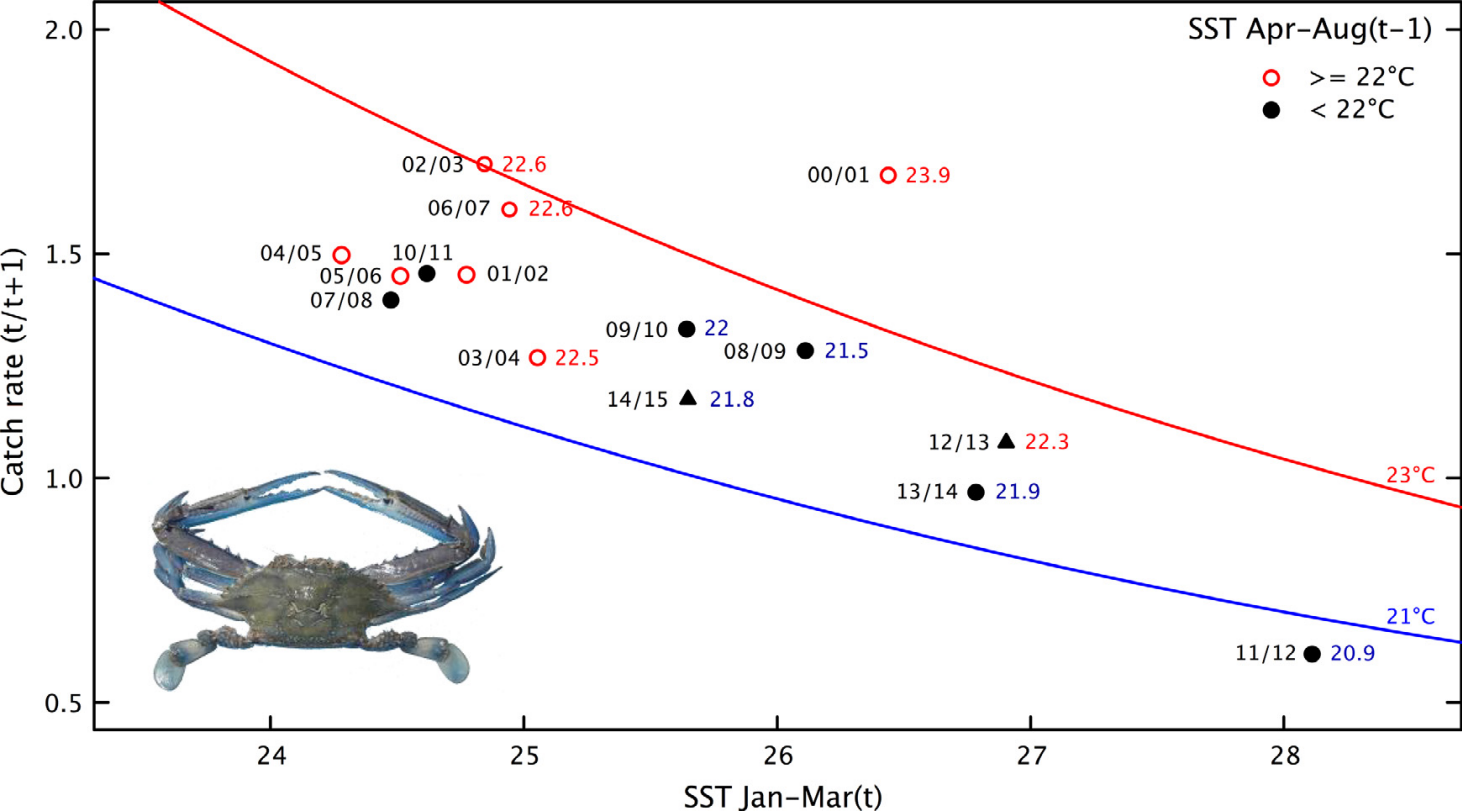
Shark Bay Blue swimmer crabs: Relationship between the annual standardized commercial trap catch rate (year *t*/*t* + 1) and mean summer sea‐surface temperature (SST) during juvenile phase and peak spawning period in the previous winter. The year of the catch rate is shown with the winter (April–August) SST. The solid triangle represents predicted catch rates for 2012/13 and 2014/15.

Spawning occurs year‐round in the tropical waters of Shark Bay (de Lestang et al. 2003; Harris et al. 2012) with a spawning peak in winter with the crabs recruiting to the fishery at the age of 1–2 years. Therefore, a correlation assessment of commercial trap catch rates and monthly SST in the previous 2 years was undertaken. This identified two key periods with significant correlations; the summer period during the juvenile phase had a negative correlation (*r* = −0.76, *P* < 0.01) and near the time of peak spawning in late autumn/winter (*r* = 0.70, *P* = 0.01). The multiple regression relationship using both these periods was highly significant (multiple *r* = 0.93, *P* < 0.001) (Fig. 4).

This indicated that warm temperatures can have a positive and negative effect on recruitment depending on the time of year they occur. Shark Bay experienced a very cool winter in 2010 followed by the heat wave in the summer of 2010/11, and this may have caused the low recruitment in 2011/12 (Fig. 4). Fishery‐independent surveys showed a partial improvement in stock in 2012/13 and 2013/14 as the winter SST in 2011 and 2012 was within historic levels but the summer SST in 2011/12 and 2012/13 remained warm (Caputi et al. 2015). Therefore, the fishery was opened in 2013/14 with a nominal catch quota of 400 *t* that was lower than the recent catches of 700–800 *t*.

An assessment of water temperature on the crab legal‐size catch rates from a fishery‐independent trawl survey showed a similar correlation pattern to that with commercial catch rates; with the SST during the juvenile period of the previous summer having a significant negative correlation (*r* = −0.89, *P* < 0.01) and a positive correlation with the winter spawning period (*r* = 0.57, *P* = 0.10).

### Scallop fisheries

The key scallop (*Amusium balloti*) trawl fisheries in Western Australia are based in Shark Bay which consists of two separate stocks; northern Shark Bay and Denham Sound (Kangas et al. 2012) and the other fishery is at the Abrolhos Islands. Trawl surveys have been conducted during November since the 1980s in Shark Bay and the late 1990s at the Abrolhos. These measure the abundance of 2 year classes, 0+ and 1+, and are used for catch prediction in the following season and in the harvest strategy control rules which determine the management arrangements (Joll and Caputi 1995).

Scallop catches in Shark Bay and Abrolhos fluctuate markedly between years and appear to be strongly influenced by water temperature with high water temperatures (associated with La Niña and strong Leeuwin Current) always resulting in poor recruitment (Joll and Caputi 1995; Lenanton et al. 2009). The initial impact of the heat wave was on the commercial catch in Shark Bay in 2011 due to poor growth and mortality with fishers noting that meat quality was poor. Management action was initiated to ensure an adequate spawning stock in the 2011 winter. However, the annual trawl survey in November 2011 resulted in a record‐low 0+ pre‐recruit and 1+ residual abundance. Hence, a proactive management approach was adopted with the fishery being closed in 2012 with industry's support.

**Figure 5:**
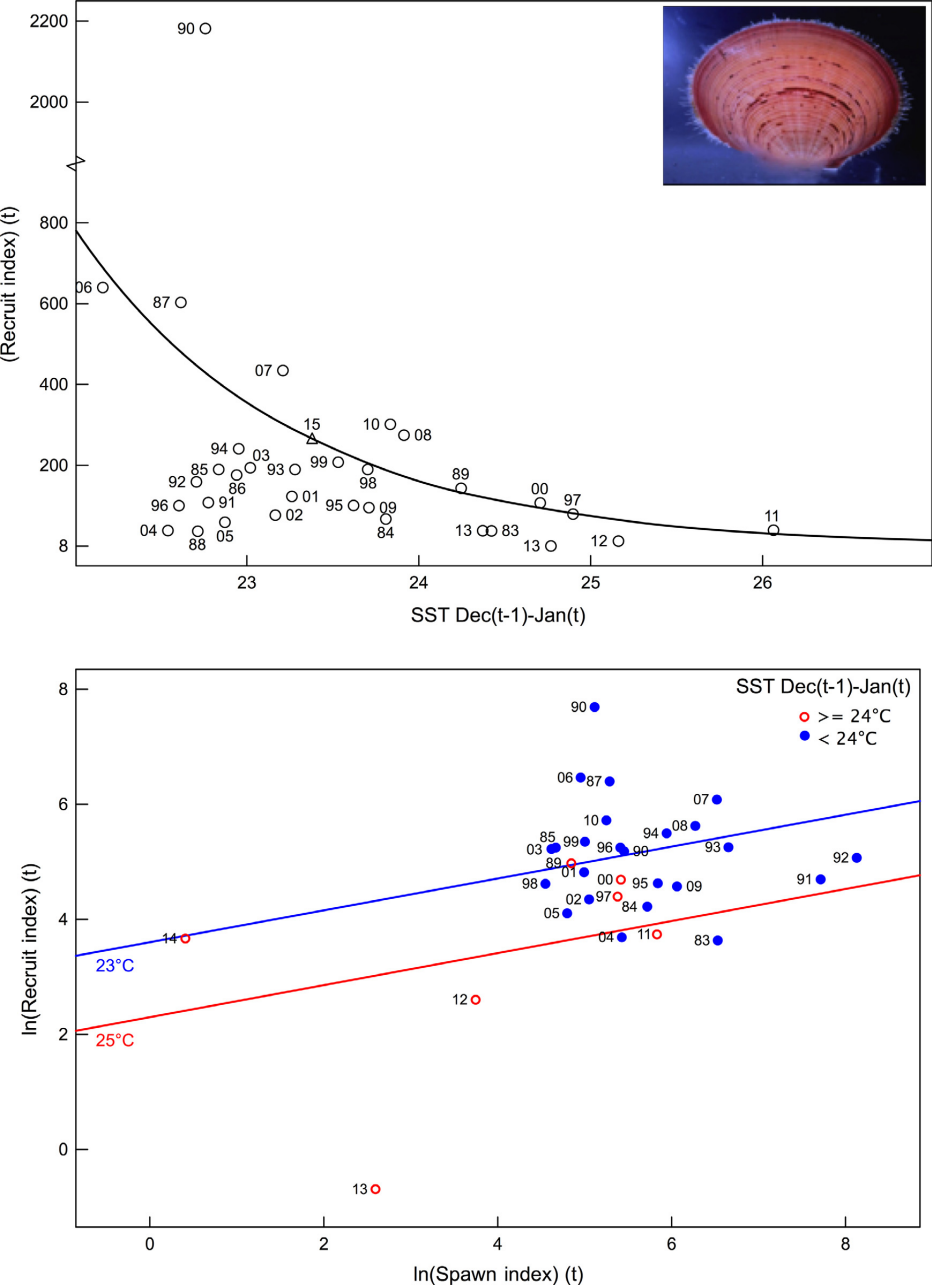
Shark Bay scallops: Relationship between pre‐recruit (0+) November survey catch rate and sea‐surface temperature (SST) in the previous summer (December–January) (top). The recruit year is shown. Relationship between the pre‐recruit index (log‐transformed) with the spawning stock index (bottom). The recruit year is shown with the points indicating whether the SST during December–January is above or below 24°C. The predicted pre‐recruit abundance for 2015 based on the spawning stock and SST is also indicated.

The relationship between the 0+ pre‐recruit catch rate in November and the monthly SST in the previous 18 months identified that warm SSTs during the summer prior to winter spawning and during the spawning period had a negative effect on recruitment (*r* = −0.46, *P* < 0.01) (Fig. 5A).

The poor 0+ pre‐recruit in November 2011 survey was due to the record warm temperatures associated with the heat wave and was expected based on previous assessments by Joll and Caputi (1995). The heat wave also affected the survival of residual stock probably associated with high mortality of adults from thermal stress. The November 2012 and 2013 pre‐recruit surveys again indicated very low abundance of 0+ and 1+ and the Shark Bay scallop fishery remained closed during 2013 and 2014.

The series of low scallop recruitment also meant that the spawning stock was reduced to historic low levels during 2012–2014. The combined 0+ and 1+ abundance from the November survey has been used as the spawning index as the 0+ pre‐recruits are generally mature by the following winter when spawning takes place. The stock‐recruitment–environment relationship showed that the spawning stock in 2012 to 2014 was in a declining trend so that the 0+ pre‐recruits in these years were influenced by historic‐low spawning stock as well as above‐average SST (Fig. 5B). Environmental conditions improved in 2014 resulting in an improved recruitment but still well below historic levels, and therefore, the fishery in northern Shark Bay will not open in 2015. However, the improved spawning stock expected for 2015 combined with cooler SSTs during the 2014/15 summer may result in an improved pre‐recruit abundance in the November 2015 survey.

Assessment of the Denham Sound and Abrolhos recruitment with SST in the previous 2 years showed a similar result in the northern Shark Bay stock with a negative effect of warm SST on the recruitment (Caputi et al. 2015). The stock‐recruitment–environment relationship also indicated that the decline in spawning stock was affecting the recruitment in both stocks. Both fisheries also showed an improved pre‐recruit abundance in 2014 with a moderate level of fishing of scallops proposed for Denham Sound in 2015.

### Shark Bay and Exmouth Gulf prawn fisheries

The Shark Bay and Exmouth Gulf prawn trawl fisheries are the two largest prawn fisheries in Western Australia and two key species are the western king prawn (*Penaeus latisulcatus*) and the brown tiger prawn (*P. esculentus*).

Spawning of both species occurs during autumn and/or spring with recruitment to the fishery in the following autumn. Recruitment has been measured in March–April in Shark Bay since 2000 for both species and in Exmouth for tiger and king prawns since the early 1980s and mid‐2000s, respectively. The recruitment abundance has been used for catch prediction and determining the management approach for the coming season (e.g., real‐time area openings based on abundance and size of prawns).

**Figure 6:**
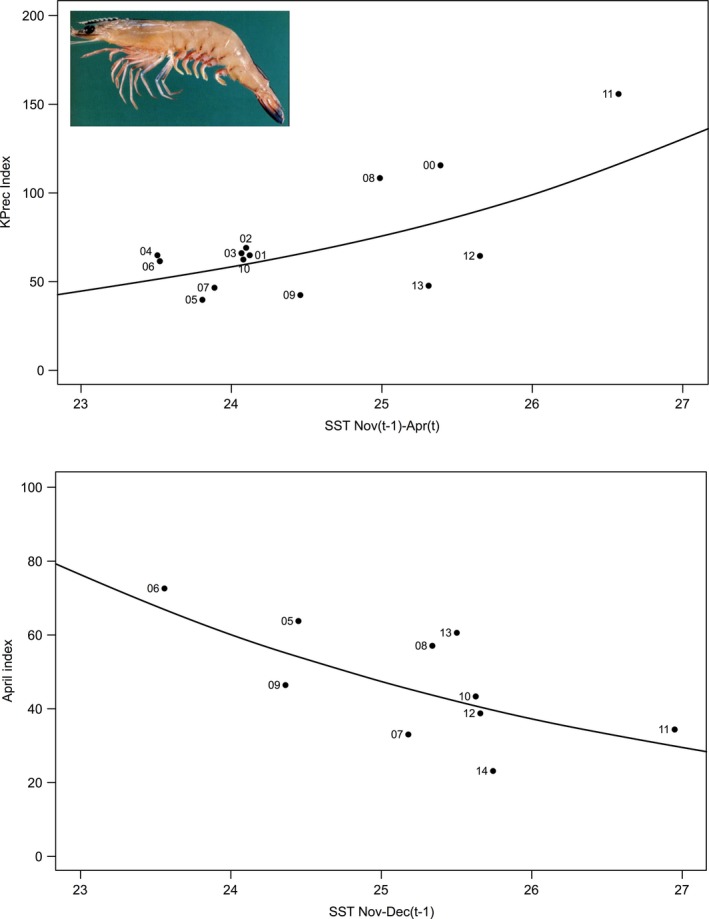
Positive and negative relationship between king prawn recruitment (year *t*) and sea‐surface temperature (SST) (November year *t*−1 to April year *t*) in Shark Bay (top) and SST (November–December in year *t*−1) in Exmouth Gulf, respectively (bottom). The recruitment year is indicated.

The tiger and king prawn recruitment index was compared with monthly SST over the previous 24 months and showed that the heat wave had some positive and negative effects on the prawn stocks in Shark Bay and Exmouth. A positive relationship between the recruitment of king and tiger prawns with SST during the larval/juvenile phase, October–April, was evident in Shark Bay (*r* = 0.63 and 0.83, respectively) with the highest recruitment recorded for king prawns (Fig. 6A) as well as tiger prawns in 2011 as a result of the heat wave. This analysis supported the positive relationship between king prawn catches and the Leeuwin Current that was recorded by Caputi et al. (1996) and Lenanton et al. (2009). However in Exmouth Gulf, water temperature had a negative effect on the king prawn recruitment (*r* = −0.62, *P* = 0.05) (Fig. 6B), which may reflect that the SST in Exmouth is generally about 2°C warmer than Shark Bay so that any increase in SST may push the SST above the optimal range.

The tiger prawn recruitment in 2012 was at a record low level, and this may be due to the heat wave causing the loss of seagrass/algae habitat in the nursery areas and this is currently being assessed. The tiger prawn recovery during 2013 and 2014 has been slow which reflects the previous experience in 2000 when a severe category 5 cyclone in 1999 caused significant physical damage to inshore nursery habitats (Loneragan et al. 2013). The tiger prawn catch slowly recovered between 2000 and 2004 as the habitats recovered.

## Discussion

The marine heat wave event in 2010/11, and the above‐average temperatures in the summer of 2011/12 and 2012/13, represented an example of an extreme environmental event in an area which is one of the warming hot spots in the world (Hobday & Pecl, 2013). Hobday and Pecl (2013) suggest that these hot spots represent frontline regions or natural laboratories to study impacts and evaluate adaptation options for marine ecosystems and fisheries. Therefore, lessons learnt in this study may be relevant to other areas affected by marine heat waves. The heat wave had a major effect on prawns, scallops, abalone, and crabs over the midwest coast of Australia in Kalbarri, Shark Bay, Exmouth Gulf, and the Abrolhos Islands and provides a case study to assess how this has affected the stocks and management of the fisheries.

The consecutive occurrences of the warming events off the Western Australian coast were observed during a global warming hiatus period, a La Niña‐like condition associated with negative Interdecadal Pacific Oscillation phase (Feng et al. 2015). Cai et al. (2015) indicate a near doubling in the future of extreme La Niña instigating marine heat wave events similar to the one in 2010/11. This highlighted the importance of assessing the effect of heat waves on fisheries. A key question was whether these events can be predicted so that an early warning system can be established. Doi et al. (2013) indicated the ability to predict Ningaloo *Niño* events 9 months in advance, although the existing climate models still underestimate the amplitude of the warming events off the West Australian coast.

Fish kills, particularly the abalone stocks in Kalbarri, were the first effect observed during the heat wave. The next key change observed was reduced growth and poor meat quality of scallops in both the Abrolhos and Shark Bay scallop fishery. Reduced growth followed by poor recruitment has also been noted in the Cockburn Sound crab fishery (D. Johnston, pers. comm.) and the Perth Roei abalone fishery (A. Hart, pers. comm.) after the heat wave. Poor growth appeared to be the first indication that the subsequent spawning stock and recruitment may be negatively affected.

The heat wave resulted in some important changes to the prawn stocks in Shark Bay and Exmouth Gulf. Tiger prawns are at the southern end of their range in Shark Bay; therefore, the warmer temperatures resulted in an improvement in stocks. A positive relationship was also evident in the recruitment of king prawns with SST in Shark Bay which supports the relationships reported previously (Caputi et al. 1996; Lenanton et al. 2009). However, heat wave with a 2–3°C spike in SST had a negative effect on the prawn stocks in Exmouth Gulf, which is on average about 2°C warmer than Shark Bay. The effect on the tiger prawns in Exmouth Gulf appeared to be an indirect effect with the heatwave affecting the seagrass habitat which has resulted in a low recruitment in 2012 and 2013. A similar effect of seagrass reduction on tiger prawn recruitment occurred in the early 2000s due to a strong cyclone in 1999 (Loneragan et al. 2013). While Fraser et al. (2014) reported a negative effect on the seagrass in Shark Bay due to the heat wave in combination with a flooding event, their study area was in the southern part of Shark Bay and does not appear to have negatively affected the prawn stocks. This highlighted the importance of monitoring changes in habitat structure for key species that are reliant on key habitats as Pratchett et al. (2011) identified that the most immediate effect of climate change may be due to changes in availability of critical habitats.

The experience of research, management, and industry in dealing with the heat wave event on the invertebrate fisheries provided some valuable lessons that are generally applicable to dealing with extreme environmental events. The pre‐recruit surveys that are conducted for the prawn, scallop, and crab fisheries that enable a prediction of future catches (Caputi et al. 2014a) have been invaluable for early detection of any change in abundance. This has enabled management and industry to adjust quickly to the lower abundance by reducing catch and effort or even the temporary closure of the fishery. Many fisheries are generally reliant on commercial catch rates to assess the status of stocks for management, so there is an inherent delay between any decline in abundance and management action which can result in the stocks being overfished during periods of low recruitment which causes a delay in their recovery.

Historical research on the relationship between fisheries recruitment and the physical environment have proved crucial for early detection of the cause of recruitment variation and for management to take immediate actions after the extreme marine heat wave event (Caputi et al. 2015). These relationships also enable the identification of species that may be at risk from warming events and climate change.

There is an increased likelihood of extreme events becoming more common as a result of climate change and decadal climate variability (Cai et al. 2015; Feng et al. 2015). Therefore, early detection of unusual environmental trends as well as stock changes becomes even more important as these changes are likely to result in the need for management intervention due to changes in stock abundance. Hobday et al. (2016) suggested real‐time monitoring using their hierarchical classification of metrics applied to daily SST data which would allow warnings to be issued as areas approached or exceeded threshold levels that would classify the event as a marine heat wave. Early detection of warming events was identified as a priority area of research after the effect of the 2012 event in the Northwest Atlantic on the American lobster fishery (Mills et al. 2013). Water temperatures are now closely monitored before the start of the lobster season and a predictive system has been developed as an early warning signal to fishers and processors on the timing of the molt which affects the start on the lobster season (http://gmri.org/news/tidings/new-forecast-sheds-light-timing-lobster-harvest).

An important aspect of early management intervention was having harvest strategies with appropriate control rules that are responsive to the changes in abundance (Department of Fisheries, 2015). When these harvest strategies also take into account the pre‐recruit abundance, then this allowed management changes before any poor year classes are fished which enhanced the protection of the spawning stock.

For the west coast of Australia, global warming has had a greater influence on the austral winter temperature whereas the marine heat waves have tended to strike during the austral summer. Thus, different climatological and oceanographic processes are involved in driving these temperature anomalies. Understanding the climatic drivers of the extreme temperature events is crucial to prediction of these events in the future. Whereas the global warming induced temperature rise will continue into the future, marine heat wave events may also be influenced by decadal climate variability. Understanding and predicting decadal climate variability is important for the immediate term fisheries management.

The marine habitats for these fisheries are also susceptible to extreme ocean temperatures. There has been some observed recovery of benthic habitats in the Abrolhos region since the 2010/11 marine heat wave (Wernberg et al. 2013); however, there is some inhomogeneity of the recovery process (Bridge et al. 2014). Thus, it is important to continue to monitor both the physical and biological environment in the region to ensure the long‐term prospect of the fisheries. The management adaptation strategies used for the Western Australian fisheries can also be applied to other regions affected by extreme events such as the strong warming events which occurred in the Northwest Atlantic (Mills et al. 2013) and Mediterranean regions (Garrabou et al. 2009). Under the Hobday et al. (2016) definition of a marine heat wave, there have been about 50–70 heat waves over the last 30 years in each of the three areas they examined as having major heat wave events in the 21st century, Western Australia, Northwest Atlantic and Mediterranean. This emphasizes the importance of having appropriate management adaptation approaches in response to these events.

## Conflict of Interest

None declared.

## References

[ece32137-bib-0001] Bond, N. A. , M. F. Cronin , H. Freeland , and N. Mantua . 2015 Causes and impacts of the 2014 warm anomaly in the NE Pacific. Geophys. Res. Lett. 42:3414–3420.

[ece32137-bib-0002] Bridge, T. C. L. , R. Ferrari , M. Bryson , Hovey, R. , W. F. Figueira , S. B. Williams , et al. 2014 Variable responses of benthic communities to anomalously warm sea temperatures on a high‐latitude coral reef. PLoS One 9:e113079. 25426718 10.1371/journal.pone.0113079 PMC4245080

[ece32137-bib-0003] Cai, W. , G. Wang , A. Santoso , M. J. McPhaden , L. Wu , F.‐F. Jin , et al. 2015 Increased frequency of extreme La Nina events under greenhouse warming. Nature Clim. Change 5:132–137, advance online publication.

[ece32137-bib-0004] Caputi, N. , W. J. Fletcher , A. Pearce , and C. F. Chubb . 1996 Effect of the Leeuwin Current on the recruitment of fish and invertebrates along the Western Australian coast. Mar. Freshw. Res. 47:147–155.

[ece32137-bib-0005] Caputi, N. , S. de Lestang , M. Feng , and A. Pearce . 2009 Seasonal variation in the long‐term warming trend in water temperature off the Western Australian coast. Mar. Freshw. Res. 60:129–139.

[ece32137-bib-0006] Caputi, N. , S. de Lestang , A. Hart , et al. 2014a Catch predictions in stock assessment and management of invertebrate fisheries using pre‐recruit abundance; case studies from Western Australia. Rev. Fish. Sci. 22:36–54.

[ece32137-bib-0007] Caputi, N. , G. Jackson , and A. Pearce . 2014b The marine heat wave off Western Australia during the summer of 2010/11 – 2 years on. Fisheries Research Report No. 250, Department of Fisheries, Western Australia. 36 pp.

[ece32137-bib-0008] Caputi, N. , M. Feng , A. Pearce , et al. 2015 Management implications of climate change effect on fisheries in Western Australia: Part 1 Environmental change and risk assessment. FRDC Project 2010/535. Fisheries Research Report 260, Department of Fisheries, Western Australia, 180 pp.

[ece32137-bib-0009] Cerrano, C. , G. Bavestrello , C. N. Bianchi , et al. 2000 A catastrophic mass‐mortality episode of gorgonians and other organisms in the Ligurian Sea (northwestern Mediterranean), summer 1999. Ecol. Lett. 3:284–293.

[ece32137-bib-0011] Department of Fisheries . 2015 Harvest strategy policy and operational guidelines for the aquatic resources of Western Australia. Fisheries Management Paper No. 271. Department of Fisheries, Western Australia, 41 pp.

[ece32137-bib-0012] Depczynski, M. , J. P. Gilmour , T. Ridgway , et al. 2012 Bleaching, coral mortality and subsequent survivorship on a West Australian fringing reef. Coral Reefs 32:233–238.

[ece32137-bib-0013] Doi, T. , S. K. Behera , and T. Yamagata . 2013 Predictability of the Ningaloo Niño/Niña. Sci. Rep. 3:2892. 24100593 10.1038/srep02892 PMC3792415

[ece32137-bib-0014] Feng, M. , M. J. McPhaden , S. Xie , and J. Hafner . 2013 *La Niña* forces unprecedented Leeuwin Current warming in 2011. Sci. Rep. 3:1277. 23429502 10.1038/srep01277 PMC3572450

[ece32137-bib-0015] Feng, M. , H. H. Hendon , S.‐P. Xie , et al. 2015 Decadal increase in Ningaloo *Niño* since the late 1990s. Geophys. Res. Lett. 42:104–112.

[ece32137-bib-0016] Fraser, M. W. , G. A. Kendrick , J. Statton , et al. 2014 Extreme climate events lower resilience of foundation seagrass at edge of biogeographical range. J. Ecol. 102:1528–1536.

[ece32137-bib-0017] Garrabou, J. , R. Coma , N. Bensoussan , et al. 2009 Mass mortality in northwestern Mediterranean rocky benthic communities: effects of the 2003 heat wave. Glob. Change Biol. 15:1090–1103.

[ece32137-bib-0018] Harris, D. , D. Johnston , E. Sporer , M. Kangas , N. Felipe , and N. Caputi . 2012 Biology and management of a multi‐sector blue swimmer crab fishery in a subtropical embayment – Shark Bay, Western Australia. Mar. Freshw. Res. 63:1165–1179.

[ece32137-bib-0019] Hart, A. 2014 The effect of the 2011 marine heat wave on the Roe's abalone (*Haliotis roei*) stocks in Western Australia Pp. 12 *in* Caputi N., Jackson G., Pearce A., eds. The marine heat wave off Western Australia during the summer of 2010/11 – 2 years on. Fisheries Research Report No. 250, Department of Fisheries, Western Australia.

[ece32137-bib-0020] Hobday, A. , and G. Pecl . 2013 Identification of global marine hotspots: sentinels for change and vanguards for adaptation action. Rev. Fish Biol. Fisheries 24:415–425.

[ece32137-bib-0021] Hobday, A. J. , L. V. Alexander , S. E. Perkins‐Kirkpatrick , D. A. Smale , S. C. Straub , and E. C. J. Oliver . 2016 A hierarchical approach to defining marine heatwaves. Prog. Oceanogr. 141:227–238.

[ece32137-bib-0022] Joll, L. M. , and N. Caputi . 1995 Environmental influences on recruitment in the saucer scallop (*Amusium balloti*) fishery of Shark Bay, Western Australia. ICES Mar. Sci. Symp. 199:47–53.

[ece32137-bib-0023] Kangas, M. I. , A. Chandrapavan , Y. L. Hetzel , and E. C. Sporer . 2012 Minimising gear conflict and resource sharing issues in the Shark Bay trawl fisheries and promotion of scallop recruitment. Fisheries Research Report No 229. Department of Fisheries Western Australia. 136 p.

[ece32137-bib-0024] Lenanton, R. C. , N. Caputi , M. Kangas , and M. Craine . 2009 The ongoing influence of the Leeuwin Current on economically important fish and invertebrates off temperate Western Australia – has it changed? J. R. Soc. West Aust. 92:111–127.

[ece32137-bib-0025] de Lestang, S. , N. Hall , and I. C. Potter . 2003 Reproductive biology of the blue swimmer crab (*Portunus pelagicus*, Decapoda: Portunidae) in five bodies of water on the west coast of Australia. Fish. Bull. 101:745–757.

[ece32137-bib-0027] Loneragan, N. R. , M. Kangas , M. D. E. Haywood , et al. 2013 Impact of cyclones and aquatic macrophytes on recruitment and landings of tiger prawns *Penaeus esculentus* in Exmouth Gulf, Western Australia. Estuar. Coast. Shelf Sci. 127:46–58.

[ece32137-bib-0028] Mills, K. E. , A. J. Pershing , C. J. Brown , et al. 2013 Fisheries management in a changing climate. Lessons from the 2012 Ocean Heat Wave in the Northwest Atlantic. Oceanography 26:191–195.

[ece32137-bib-0029] Pearce, A. , and M. Feng . 2007 Observations of warming on the Western Australian continental shelf. Mar. Freshw. Res. 58:914–920.

[ece32137-bib-0030] Pearce, A. F. , and M. Feng . 2013 The rise and fall of the “marine heat wave” off Western Australia during the summer of 2010/2011. J. Mar. Syst. 111–112:139–156.

[ece32137-bib-0031] Pearce, A. , R. Lenanton , G. Jackson , et al. 2011 The “marine heat wave” off Western Australia during the summer of 2010/2011. Fisheries Research Report 222, Department of Fisheries, Western Australia, 36 pp.

[ece32137-bib-0033] Pratchett, M. S. , L. K. Bay , P. C. Gehrke , et al. 2011 Contribution of climate change to degradation and loss of critical fish habitats in Australian marine and freshwater environments. Mar. Freshw. Res. 62:1062–1081.

[ece32137-bib-0034] Reynolds, R. W. , T. M. Smith , C. Liu , D. B. Chelton , K. S. Casey , and M. G. Schlax . 2007 Daily high‐resolution‐blended analyses for sea surface temperature. J. Clim. 20:5473–5496.

[ece32137-bib-0035] Smale, D. A. , and T. Wernberg . 2009 Satellite‐derived SST data as a proxy for water temperature in nearshore benthic ecology. Mar. Ecol. Prog. Ser. 387:27–37.

[ece32137-bib-0037] Wernberg, T. , D. A. Smale , F. Tuya , et al. 2013 An extreme climatic event alters marine ecosystem structure in a global biodiversity hotspot. Nat. Clim. Chang. 3:78–82.

